# Recent onset mental illness severity: pilot study on the role of cognition, sensory modulation, and daily life participation

**DOI:** 10.3389/fpsyt.2024.1413635

**Published:** 2024-09-30

**Authors:** Lena Lipskaya-Velikovsky, Ayelet Hershkovitz, Mira Bukai, Tami Bar-Shalita

**Affiliations:** ^1^ School of Occupational Therapy, Faculty of Medicine, The Hebrew University, Jerusalem, Israel; ^2^ The Jerusalem Mental Health Center, Jerusalem, Israel; ^3^ Department of Occupational Therapy, School of Health Professions, Faculty of Medical and Health Sciences, Tel-Aviv University, Tel-Aviv, Israel

**Keywords:** objective participation, subjective participation, cognitive failures, cognitive biases, sensory processing, serious mental illness, intensive care

## Abstract

**Introduction:**

Early detection of individuals at risk for onset of severe illness is crucial for prevention and early intervention, aiming to mitigate the long-term impact on both the individual and the community. While well-established models exist for predicting the onset and prolonged severity of illness, there is a gap in understanding illness-onset severity. This pilot study aimed to investigate premorbid objective and subjective dimensions of participation in daily life occupations, as well as sensory and cognitive functions as potential markers of the recent-onset mental illness severity.

**Methods:**

A total of 50 participants (men: N=26, 52%; women: N=24, 48%), aged 18–40 (M=26.2, SD=5.8) with recent-onset mental illness completed standard, well-established assessments of illness severity, cognitive biases and failures, neurocognitive status, participation in daily life, and sensory responsiveness thorough cross-sectional design. The differences between the groups of the illness severity were explored with descriptive statistics, followed by a Kruskal–Wallis test. Discriminant analysis was used suggesting a multi-varied model for the separation between the groups of illness severity.

**Results:**

Three groups of illness severity exhibited differences in premorbid cognitive functions (F(2)=5.8, p<.01) and participation diversity (F(2)=3.8, p<.05). Combining these two indices explained 92% of the variance between the groups (Wilks’ Λ = .68, χ2(4) = 17.7, p=.001), accurately classifying mild to marked illness severity (62.5–88.5%).

**Conclusions:**

The study contributes to revealing factors involved in the formation of more severe mental illness and suggesting possible avenues for early intervention and prevention. Cognitive biases and sensory modulation dysfunction may contribute to the illness formation. Still, the most effective markers of more severe mental illness onset are functional cognition and limited participation diversity. Since addressing these markers is a unique specialization within occupational therapy, the findings highlight the potential contribution the profession can make to the early identification of the most vulnerable populations.

## 1 Introduction

Mental illness onset can be a life-changing event for individuals and their loved ones, raising significant uncertainty about the future (1, 2). Indeed, it was demonstrated that mental illness affects well-being and general health indices, disrupts autonomy, choice, and feeling of being in control, interrupts a positive self-image and a sense of belonging, intervenes with engagement in meaningful and enjoyable activities, and challenges the experiences of hope and optimism (3). The illness severity at early stages is as important as being a predictor of future illness trajectory and general health outcomes (4–7). Interventions for those who are at risk of developing more severe illness have the potential to eliminate the long-term burden of mental health conditions (1, 2). Thus, identifying markers for the development of more severe illness can inform both preventive strategies and early interventions, which should be tailored to individual needs (1, 2). Moreover, such understanding could bridge the gap between models predicting illness onset (2) and those explaining long-term outcomes of mental illness (4, 8).

While there is extensive research on illness onset (2, 5) and prolonged mental illness severity (9, 10), models for explaining illness severity in recent-onset are scarcely studied. The staging approach suggests a vision of mental illness as a continuum with shared neurobiological and psychosocial underlying mechanisms through illness formation, onset, progression, and persistence (5). Thus, some similarities may be assumed between markers for the transition to illness, and illness severity at different stages. Moreover, recent illness onset is a particular challenge, since it is frequently characterized by shared signs and symptoms through various diagnoses (e.g., psychosis, depression, anxiety, etc.), multiple changes in symptom presentations, and switches between given diagnoses over time (5, 11). Thus, a transdiagnostic approach that distills similarity in neurobiological, genetic, and psycho-social processes underlying mental illness across the boundaries of diagnoses is of relevance (5, 12, 13).

In the context of the transition into mental illness, it was demonstrated that genetic factors, previous level of functioning, neurocognitive deficits, and aberrant thinking patterns (2, 5, 6, 14–16), as well as personal factors such as age, gender, years of education, history of trauma, and substance use (2, 5) may serve as precursors of a range of mental health disorders. The prediction of long-term outcomes from the illness onset was found to be based on premorbid employment status and educational level (6, 14), general functional status at baseline (6, 14, 17), cognitive level (6, 8, 17), social support, adverse life events, illness duration, duration of untreated psychosis, treatment adherence, hospitalization, and demographic factors (8–10, 14). Thus, to make an initial step toward model building, this pilot study was designed to investigate shared factors between the illness stages as possible markers of recent-onset illness severity. The study also innovatively suggests additional markers based on the theoretical background of occupational therapy regarding factors promoting health (18), as well as general, intradisciplinary models of health, disability and functioning (19).

### 1.1 Cognition

Neurocognitive deficits which include processing speed, attention, learning and memory, problem-solving, and working memory are hallmarks of prolonged mental illness (3, 7). These have been reported before the illness onset, in early childhood (15, 20), and serve as a precursor of mental illness onset (6, 8, 15, 17). Being independent of illness symptoms, neurocognitive deficits have been suggested to be involved in illness formation and persistence.

An additional construct of interest in the field of mental illness is the non-functional thinking patterns―cognitive biases. Being distinct from neurocognitive functioning (16, 21), aberrant thinking patterns about the world and events include anomalous perception with attribution and over-interpretation of events as threatening, catastrophic, and dichotomous thinking, jumping to conclusions, and emotionally-based reasoning (22). These thinking patterns elicit inaccurate judgment and unusual insights into the reality underlying some mental illness signs (16, 21, 23). The cognitive biases can also manifest within the context of social situations. Attribution biases refer to idiosyncratic patterns of comprehension and interpretation of social events and interactions (e.g., blaming others for negative occurrences). These biases were found to be one of the core domains of social cognition and impaired in schizophrenia (24). With their developmental trajectory (22), aberrant thinking patterns were reported in individuals with resent-onset psychosis (16, 23), but also a core element in major depression and anxiety (25). However, the literature in the field of early illness onset is inconclusive; for example, no differences were found between individuals with early psychosis and healthy controls in the attribution biases within social context (26).

### 1.2 Sensory modulation

Recent literature suggests that alterations in sensory modulation play a significant role in mental illness (12). Sensory modulation is a process in which the brain interprets sensory information of all modalities to produce context-appropriate behavioral and emotional responses meeting individual needs (27–29). Interference in sensory modulation―sensory modulation dysfunction (SMD)―has been found with a prevalence of 5–18% in the general population, escalating 2–4 times in schizophrenia, affective disorders, and anxiety (27, 28). Different types of SMD were detected representing typical patterns of association between an altered interpretation of sensory input and following, idiosyncratic behavioral and emotional responses, mainly maladaptive and disproportional (27–29). Originally guided by behavioral representation, the assumption of alterations in sensory processing received support from electrophysiological and imaging studies in various mental health conditions (30). It was demonstrated that in otherwise healthy young adults, SMD substantially affects an individual’s life, psychological well-being, and everyday functioning (31).

### 1.3 Participation in daily life occupations

Most research has investigated objective aspects of general functioning in everyday life in recent-onset and prolonged illness (7, 8, 14, 32, 33) or has been specific to employment and education status (6, 14). The World Health Organization (19) put everyday functioning in a broader concept of participation―an involvement in life situations, emphasizing the need to address a range of everyday life occupations (19). Focused literature on participation further expands the concept, arguing for its complex nature encompassing objective dimensions of attendance at activities (aspects of everyday functioning) but also subjective dimensions of the experience of involvement (34). Indeed, it was found that subjective dimensions of participation, which were mostly omitted in the research to date, are of particular importance to well-being in mental health (35). Moreover, most studies have focused on objective measures of functioning at baseline or after the onset of mental illness (8, 14, 32, 33), limiting our ability to investigate the predictive quality of everyday functioning alterations for understanding the trajectory of mental illness.

### 1.4 Rationale and study aim

Mental illness may have a pronounced impact on the person and the whole community life, while serious mental illness substantially interferes with one or more major life activities, such as work, home management, and social relationships (4, 8). Identifying early signs of illness severity is crucial for tailoring prevention and intervention strategies, aiming to mitigate the enduring impact of mental illness. Thus, this study was designed to investigate the feasibility of building an explanatory model of the recent-onset illness severity, addressing a range of premorbid potential precursors. Even though there is an ongoing debate on how to conceptualize illness severity (e.g., number of symptoms, their frequency, persistence, functional disability, or quality of life) (36), for this study it was operationalized through general psychiatric symptomology. Specifically, we investigated premorbid objective and subjective dimensions of participation in daily life occupations, sensory processing, and cognitive functions as markers of the recent-onset mental illness severity. Relying on previous knowledge in the field of mental health, this study is grounded on the International Classification of Functioning, Disability, and Health (ICF) model (19) and occupational therapy models (18). This study was designed to provide insights into the onset of mental illness by addressing symptom severity through an occupational therapy perspective. This perspective posits that engagement in occupations is a crucial component of health with a mutual bi-directional relationship, and, it is a result of a dynamic interplay between personal factors, occupations, and the environment (18). Additionally, the study incorporates tools developed within occupational therapy to reflect its unique areas of concern and expertise, addressing both the objective and subjective dimensions of participation across broad areas of occupation and sensory modulation. Recent onset illness severity is important for future illness trajectory, including general health outcomes, well-being, and daily life participation (4–7). The recent onset of mental illness is a sensitive situation that requires careful consideration to determine the appropriate treatment intensity. Inadequate treatment may fail to meet the individual’s needs, potentially leading to the development of a chronic condition. Conversely, overly intense treatment may discourage individuals who may already be ambivalent about mental health interventions and exceed the resources of the health service. Identifying a population at risk can facilitate targeting those who need of more intense intervention, including, for example, occupational therapy intervention. Thus, the aim of this pilot study was to investigate premorbid objective and subjective dimensions of participation in daily life occupations, as well as sensory and cognitive functions as potential markers of the recent-onset mental illness severity. The results of the study may help to distill the role of the occupational therapy within mental health workforce acting for prevention and early intervention. These by revealing the contribution of information on participation patterns and sensory modulation―areas of professional proficiency―to the early detection of the most vulnerable population with recent mental illness onset. Understanding factors enabling early identification may expand the knowledge on the mechanisms of illness formation thereby offering possible avenues for prevention and early intervention with this population.

## 2 Methods

### 2.1 Study design

This cross-sectional study involved people with recent-onset psychiatric illness who were recruited through convenience sampling.

### 2.2 Participants

Fifty participants (men: N=26, 52%; women: N=24, 48%), aged 18–40 (M=26.2, SD=5.8) with recent-onset mental illness were recruited from intensive services of two regional mental health centers (differing geographically). For this study, recent-onset was defined as a first-time formal diagnosis of any psychiatric disorder illness based on the ICD-10 criteria. The diagnosis was validated at discharge. The participants were admitted either to secured wards (N=23, 46%), open wards (N=13, 25%), or intensive day-care programs (N=14, 26.9%), received stable medication for at least two weeks at recruitment and were admitted for less than 12 weeks (inclusion criteria). The median duration of the treatment in days was 29 (IQR: 18–52). Individuals who had previous contact with psychiatric services (except for contact in the last six months which did not lead to a diagnosis), and had a history of previous use of psychiatric medication and substance use disorder were excluded from the study. In addition, people with neurological and physical health conditions that limit participation in daily occupations and cognitive functioning were excluded from the study.

Diagnoses included psychotic spectrum disorders (N=25, 50%), affective disorders (N=19, 38%), anxiety disorders (N=3, 6%), and personality disorder (N=3, 6%). The participants had an average of 12.4 years of education (range 8–16, SD=1.8), were mostly unemployed, and lived in urban areas (**Table 1**).

**Table 1:** Demographic and illness-related data by illness severity groups (N=50).

	Minor (N=16)	Mild (N=26)	Moderate and marked (N=8)	Between-groups differences	
Gender					
Man	7 (43.8%)	17 (65.4%)	2 (25%)	χ^2^ (2)=4.64	
Woman	9 (56.3%)	9 (34.6%)	6 (75%)		
Place of living					
Urban	15 (93.8%)	23 (88.5%)	6 (75%)	χ^2^ (2)=5.47	
Not-urban	1 (6.2%)	3 (11.5%)	2 (25%)		
Job in past 6 months					
Yes	6 (37.5%)	13 (50%)	2 (25%)	χ^2^ (2)=1.77	
No	10 (62.5%)	13 (50%)	6 (75%)		
Diagnosis at discharge					
Psychotic disorders	6 (37.5%)	15 (57.7%)	4 (50%)	χ^2^ (6)=5.3	
Affective disorders	9 (56.3%)	7 (26.9%)	3 (37.5%)		
Anxiety	0	2 (7.7%)	1 (12.5%)		
Personality disorders	1 (6.3%)	2 (7.7%)	0		
Type of service					
Day treatment	5 (31.3%)	7 (26.9%)	2 (25%)	χ^2^ (4)=5.76	
Open ward	1 (6.3%)	10 (38.5%)	2 (25.5%)		
Close ward	10 (62.5%)	9 (34.6%)	4 (50%)		
	Median (Range)	Median (Range)	Median (Range)	Statistics	ES
Age	26 (21.5–30)	26.5 (22.75–30.25)	22 (20–27.75)	H (2)=2.1	.05
Education (years)	12 (11.25–14.75)	12 (12–13.25)	12 (12–12.75)	H (2)=0.02	0
In-patient staying duration (days)	21 (16–36.5)	34.5 (20.5–55.25)	27 (24–54.5)	H (2)=3	.05

Sample size calculation was based on the study of Torgalsbøen et al. (16). using an association between neurocognitive general status at baseline in the first episode of schizophrenia and general functioning at 6 months as an indicator of illness outcomes. Based on the reported correlation coefficients 0.3<r<0.5, the minimal number of participants in the study was defined as N=26 with α = 0.05 and a power of 0.85 (G*Power software). Since the current study was intended to address a range of diagnosis and explanatory factors, we doubled the sample size (N=50).

### 2.3 Measurements

To meet the study aims, we measured illness severity, premorbid cognitive functioning, thinking patterns, sensory modulation, objective and subjective dimensions of participation in a range of daily-life occupations, and current neurocognitive status.

#### 2.3.1 Illness severity

*Clinical Global Impression (CGI)* (37) is a commonly used tool for evaluating mental illness severity based on clinician reports. The illness severity sub-scale (CGI-S) is rated on a 7-point scale ranging from 1 (“normal, not at all ill”) to 7 (“among the most extremely ill patients”), based on clinical judgment and experience with the same psychiatric conditions. The CGI-S was found to be sensitive to many diagnoses (38). Its validity was demonstrated compared to well-established psychiatric tools such as Brief Psychiatric Rating Scale (0.41<r<0.74), Positive and Negative Syndrome Scale (0.54<r<0.68), and Hamilton Rating Scale for Depression (0.79<r<0.86). In addition, inter-rater reliability (r = 0.66) was reported (39).

#### 2.3.2 Sensory modulation

Sensory Responsiveness Questionnaire- Intensity Scale (SRQ-IS) (29) was used to evaluate sensory modulation patterns as a trait. This self-report questionnaire addresses the intensity of responses to daily life non-painful sensations based on predefined daily life scenarios. The measure consists of 58 statements, involving each sensory stimulus in one of the following modalities: auditory, visual, gustatory, olfactory, vestibular, and somatosensory stimuli excluding pain. The items are worded in a manner attributing a hedonic or aversive response to the sensory scenario. The participants are required to rate the intensity of the response to the stimulus described on a Likert scale ranging from 1 (“not at all”) to 5 (“very much”). The SRQ intensity scale elicits two scores: SRQ-Aversive (32 items) and SRQ-Hedonic (26 items) (29). Scores are calculated as means in each scale. Identifying SMD via one or both scales is based on normative data cut-off scores, indicating sensory over-responsiveness (SOR) or sensory under-responsiveness (SUR) (40). The SRQ has been demonstrated to have content, discriminant, criterion, and construct validity, as well as internal consistency (Cronbach α= 0.90–0.93) and test-retest reliability (r= 0.71–0.84; *P* < 0.001–0.005) (29).

#### 2.3.3 Cognitive functioning

Montreal Cognitive Assessment (MoCA) (41) was used to evaluate current neurocognitive status. The MoCA is a brief screening tool, widely used in research and clinics, aimed to assess mild cognitive impairment based on the following cognitive functions: executive functions, visuospatial abilities, short-term memory, language, attention, concentration, and working memory; and temporal and spatial orientation. A maximum score of 30 points indicates intact neurocognitive functioning (41). The test has high test-retest reliability (r =0.92, p<0.001), internal consistency (Cronbach alpha=0.83), and criterion validity, which was established in comparison to MMSE (r=0.87, p<0.001) (41).

Cognitive Failure Questionnaire (CFQ) (42) and Cognitive Biases Questionnaire (CBQ) (22) were used to investigate premorbid cognitive functions. The CFQ is a self-report questionnaire that consists of 25 items that address cognitive errors or lapses in perception, attention, memory, and motor action through everyday life activities. The responder is asked to rate the frequency of occurrence using a 5-point Likert scale (0 – Never, 4 – Very often). The highest score (100 points) represents the lowest occurrence of cognitive failures. Given the type of activities covered by the CFQ, which reflect prolonged situations in an individual’s everyday life (e.g., forgetting medical appointments and leaving important letters unattended for days), we used this measure as an index of premorbid cognitive functioning in daily life (43). The CFQ has sufficient test-retest reliability (0.80<r<0.82), internal consistency (Cronbach’s alpha = 0.91), and construct validity (44). The CBQ is a self-report questionnaire aimed to identify five types of thinking distortions: Jumping to Conclusions (JTC), Attribution, Dichotomous Thinking, Catastrophizing, and Emotionally Based Reasoning (22). The questionnaire consists of 30 statements that describe everyday situations, equally divided into two themes: Anomalous Perception (AP) and Threatening Events (TE). The participant is asked to imagine him/herself in each situation and choose the option that best describes his/her patterns of thinking about the situation. The scoring ranges from 1 (absence of bias) to 3 (likely presence of bias). The CBQ has test-retest reliability (r=0.96), internal consistency (Cronbach’s alpha = 0.89), and construct validity based on comparison with the Cognitive Style Test (0.77–0.85) (22).

#### 2.3.4 Participation patterns

Adult Subjective Assessment of Participation (ASAP) (45) is a self-report questionnaire aimed to evaluate participation patterns in everyday life activities by the following dimensions: diversity, intensity, satisfaction, enjoyment, with whom the occupations occur and where. The questionnaire addressed 52 activities, organized into 9 categories. In this study, we collected data on the objective participation dimensions of the participation diversity and intensity, as well as the subjective participation dimension of enjoyment. The data on these participation dimensions was collected as follows: the participation diversity was measured by the number of activities participated in, the intensity was calculated based on the reported frequency of actual participation in activities (7-point scale: 1 – once time within the period; 7 – several times a day; and 2 additional ratings for (a) activities that have not done before and not doing currently, and (b) activities that have done in the past, but not doing currently), and enjoyment―rating of the subjective experience on a 6-point Likert scale (0 – did not enjoy, 5 – enjoy very much). The participants were asked to report on the participation during 4 consequent routine months in 5 target occupation categories: domestic life, recreational activities, entertainment, educational activities, leisure and sports activities, and quiet recreation. Test-retest reliability ranges from 0.553–1 for different categories. Exploratory factor analysis approved a factor solution indicating sufficient construct validity. In addition, the ASAP was found to discriminate between groups of individuals with different types of health conditions and the control group (45).

### 2.4 Procedures

The Institutional Review Board of two regional mental health centers approved the study (0042-15-GEH and 102-16/4-14). The research team which consists of 2 occupational therapists approached individuals who met the inclusion and exclusion criteria. Those who agreed to participate in the study and provided written informed consent to participate following an explanation of the study’s aims and procedures were enrolled. The study procedures lasted 90 minutes, which could be divided into two shorter sessions over three days, according to the participants’ will. The participants completed a demographic questionnaire first, followed by questionnaires in a counterbalanced order: the SRQ, ASAP, CBQ, and CFQ. Finally, the MoCA test was administered. The CGI was completed by the research team (two occupational therapists) concurrently with the administration of the other instruments. The team conducted a session to establish inter-rater reliability, during which they rated five subjects and achieved an agreement level of 90%”.

The research team of 2 occupational therapists approached individuals who met the inclusion and exclusion criteria. The CGI was completed by the research team who were previously trained for its completion and conducted session.

### 2.5 Data analysis

Descriptive statistics was used to characterize the study participants for their demographic data and the study variables. Based on the SRQ scores, the study participants were classified as having SMD of hedonic type, SMD of aversive type, and non-SMD.

The data distribution was explored with the Shapiro–Wilks test, indicating normal distribution for all the measurements except for the CBQ total score. The Pearson correlation coefficient was used to investigate the association between the variables. Differences between the groups of the illness severity in demographic data and CBQ total score were explored with the Kruskal–Wallis test due to the type of data distribution. Other between-group differences were analyzed with one-way ANOVA. In addition, the effect size metric (η^2^) was calculated for all comparisons. Discriminant analysis, a multivariate statistical technique, was used to investigate the best combination of independent variables explaining the separation between the groups of illness severity. The separation is measured by the distance between the means of the groups and their variance. The accuracy of the best-fit discriminant function is represented through various statistic parameters including classification rates. Such a function may be used as a model for predicting the group membership of a new observation (46). We applied discriminant analysis with a stepwise method to build the model. The independent variables for the discriminant model were selected based on the results of the foregoing analyses. The data was analyzed using SPSS-28 (IBM) and the level of statistical significance was set at.05.

## 3 Results

### 3.1 Illness Severity and associated factors

The illness severity, as measured with CGI-S, varied among the study participants from minor mental illness (N=16, 32%), mild illness (N=26, 52%), moderate illness (N=7, 14%) up to marked illness (N=1, 2%) with Median= 3 (IQR: 2–3).

The correlational analysis indicates an association between the level of psychiatric illness and diversity of participation (r=-0.33, p<.05). The higher the level of symptomology after illness onset the lower the diversity of participation that preceded the illness onset. In addition, we found a correlation between illness severity and specific cognitive biases due to anomalous thinking (r=0.38, p<.01) and emotional reasoning (r=0.288, p<.05), even though no association was found with the total CBQ score (r=0.11, p>.05). Higher scores of specific cognitive biases were associated with a higher level of symptomology. No correlation was found with additional demographic factors, participation dimensions, MoCA score, cognitive failures, and sensory modulation indices (-0.15<r<0.24, p>.05).

**Figure 1:**
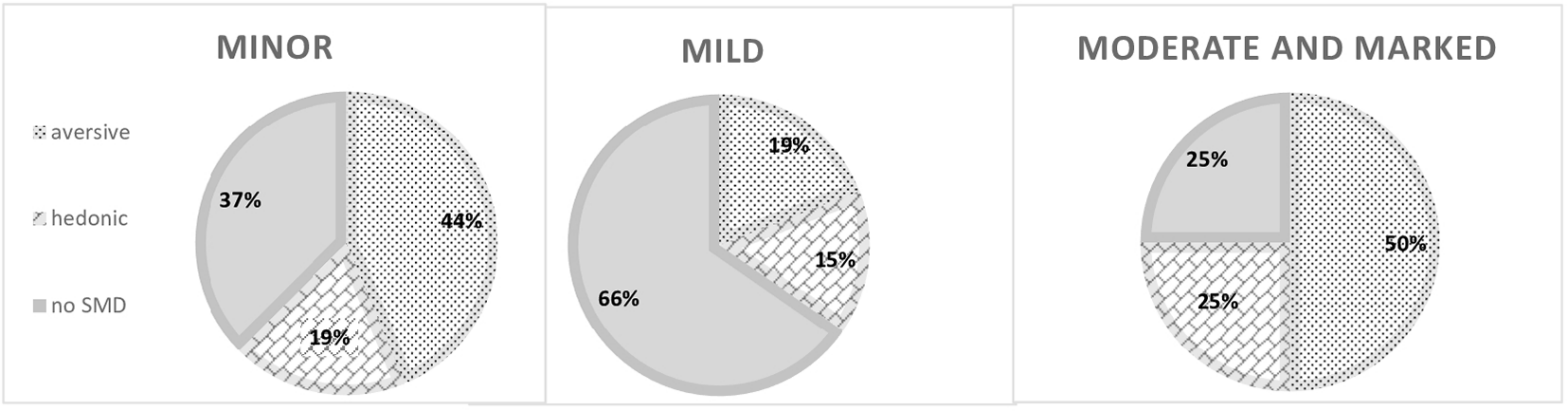
Sensory modulation pattern distribution in the minor, mild, and moderate, and marked illness groups. SMD, sensory modulation dysfunction.

For an in-depth investigation of illness severity, we combined participants with moderate and marked illness severity into one group. No differences were found between the three groups of illness severity (minor, mild, and moderate and marked) in demographic and illness-related variables (**Table 1**). However, the groups differed in the premorbid cognitive functioning as measured by the CFQ and in the diversity of participation (**Table 2**). *Post hoc* analysis revealed differences in CFQ scores between the mild and moderate/marked illness groups while the source of the difference in the participation score is a discrepancy between the moderate/marked illness group and each one of the two other groups. No differences were found in current cognitive status, cognitive biases, or participation frequency and enjoyment (**Table 2**). In addition, no statistically significant difference was found between the three illness severity groups in the sensory modulation type distribution (SMD versus non-SMD: χ^2^(2)=5.46, p=.065; SMD by types: χ^2^(4)=5.9, p=.21) (**Figure 1**). Of note, based on the ES metrics, the trend for the difference was demonstrated in the premorbid cognitive biases of dichotomous thinking and emotional-based reasoning, as well as in premorbid hedonic and aversive sensory patterns (**Table 2**).

**Table 2:** Participation, cognition, and sensory modulation indices by illness severity groups (N=50).

	Minor (N=16)	Mild (N=26)	Moderate and marked (N=8)	Between-groups analysis		
	Median (Range)/ M(SD)	Median (Range)/ M(SD)	Median (Range)/ M(SD)	Statistics	p.value	Effect Size
Cognitive measurements						
MOCA	25.5 (3.6)	24.9 (3.9)	23.7 (3.9)	F (2)=0.5	.62	.02
CFQ	48.7 (18)	39.5 (13.9)	60.25 (16.1)	F (2)=5.8^**^	.006	.2
CBQ						
Total Score	40 (37–44)	43 (39–47)	48 (39–55)	H (2)=3.2	.51	.03
Threatening events	23.3 (6.3)	22.6 (4.5)	25.6 (6.9)	F (2)=0.8	.46	.04
Anomalous perception	19.9 (4.2)	20.4 (3.4)	21.6 (2.4)	F (2)=0.5	.6	.02
Attribution	8.1 (1.5)	8.1 (1.45)	7.4 (0.8)	F (2)=0.7	.51	.03
Catastroph.	8.7 (2.4)	8.4 (1.7)	9 (1.6)	F (2)=0.3	.73	.01
Dichotomous thinking	8.1 (2.2)	7.9 (2)	10.1 (1.95)	F (2)=3.1	0.055	.12
Jumping to conclusions	10 (3)	9.8 (2.5)	10 (2.5)	F (2)=0.02	0.99	.00
Emotionally based reasoning	8.3 (2.7)	8.6 (1.8)	10.6 (2.8)	F (2)=2.5	.095	.09
Sensory modulation aspects – SRQ						
SRQ- Aversive	2.3 (0.46)	2 (0.47)	2.4 (0.73)	F (2)=1.9	.19	.08
SRQ- Hedonic	2.3 (0.54)	2.2 (0.54)	2.7(.74)	F (2)=2.3	.062	.1
Participation dimensions – ASAP						
Diversity	19.7 (8.8)	18.9 (8.7)	9.9 (9)	F (2)=3.8^*^	.029	.14
Intensity	3.4 (1.2)	3.2 (1.65)	2.8 (2.5)	F (2)=0.4	.7	.02
Enjoyment	4.5 (0.64)	4.7 (0.8)	4.8 (1.4)	F (2)=0.56	.57	.03

*p<.05; **p<.01; ASAP, Adults Subjective Assessment of Participation; CBQ, Cognitive Bias Questionnaire; CFQ, Cognitive Failure Questionnaire; MOCA, Montreal Cognitive Assessment; SRQ, Sensory Responsiveness Questionnaire.

**Figure 2:**
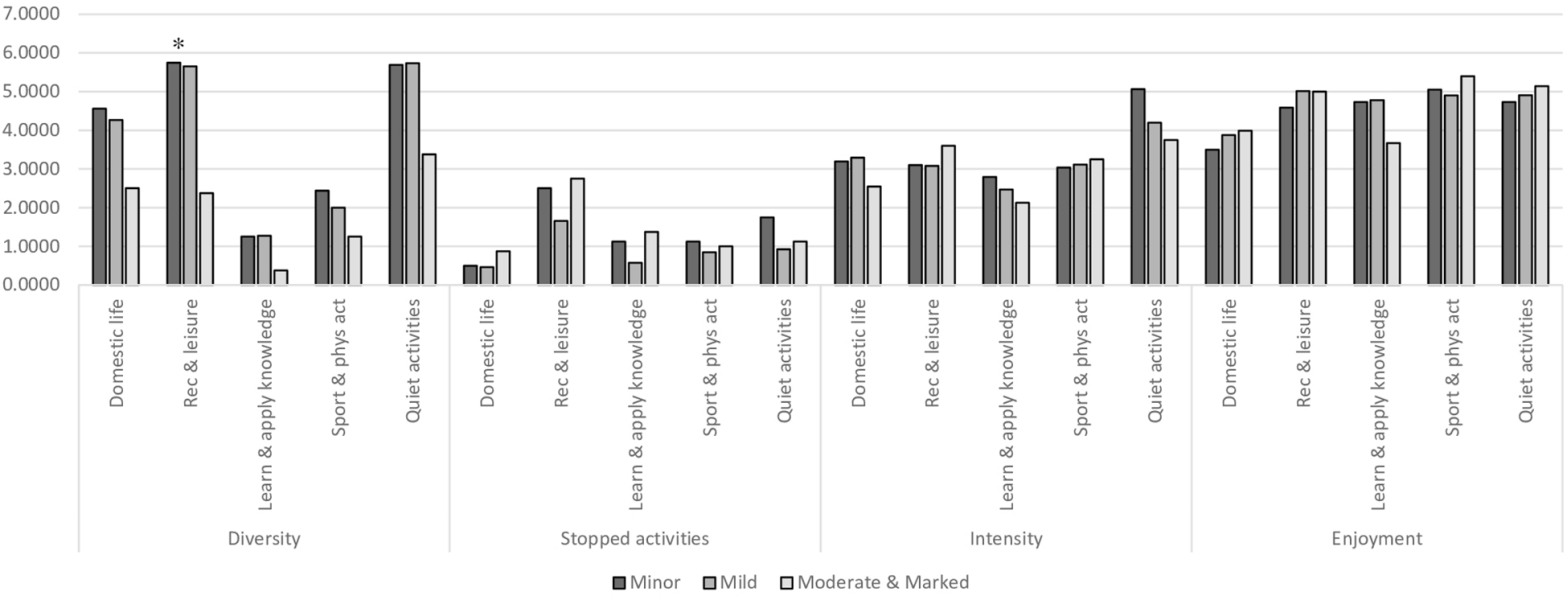
Participation dimensions by areas of occupations in study groups (the ASAP score) (N=50). ASAP, Adults Subjective Assessment of Participation; *p<.05.

The analysis of differences by areas of occupations before illness onset revealed similar patterns of participation between the groups by the illness severity (0.1<H(2)<4.2, p>.05), except for diversity of recreation and leisure activities (F(2,47)=3.8, p<.05). The results stem from the difference between both groups of milder illness severity, where participation diversity was higher, and the group of moderate and marked severity, according with *post-hoc* analysis (**Figure 2**). ES metrics suggest possibility for a similar trend of lower participation diversity in domestic life activities, learning and applying knowledge activities, and quite leisure activities among participants with moderate and marked severity (0.8< η^2^<1). In addition, effect size metrics suggest a trend for difference in several areas where the activities were stopped, i.e., quiet leisure and learning and applying knowledge (0.5< η^2^<0.8), and enjoyment in these areas (0.6< η^2^<0.8) (**Figure 2**), while those with minor illness tend to report experiencing lower enjoyment and more terminated activities.

Secondary analysis, comparing the two main types of diagnosis: psychotic spectrum disorders (N=26, 50%) and affective disorders (N=14, 26.9%). We found statistically significant differences in SRQ aversive score (U=75, p<.01) and CFQ score (U=91, p<.05) solely. Specifically, individuals with affective disorders reported higher scores on the SRQ-Aversive scale (Psychotic spectrum: Median=1.9, IQR: 1.7–2.1; affective disorders: Median=2.4, IQR: 2.1–2.9) and higher frequency of cognitive failures (Psychotic spectrum: Median=40.5, IQR: 31.3–43.8; affective disorders: Median=47.5, IQR: 36–62.8).

### 3.2 Illness severity — explanatory multivariate analysis

A two-step discriminant analysis was performed using two independent variables that showed statistically significant differences between illness severity groups: cognitive failures (CFQ) and participation diversity. Additionally, the SRQ hedonic score was included in the regression model due to its high effect size for the group differences, even though it was not statistically significant. The first step includes CFQ (Wilks’ Λ = .8, *p*=.006) and the second step includes, in addition, the participation diversity (Wilks’ Λ = .68, *p*=.001). The final discriminant function within two measurements (CFQ and participation diversity) was significant (Wilks’ Λ = .68, χ^2^(4) = 17.7, *p*=.001) explaining 92% of the variance of the latent variables within a discriminant function (canonical correlation is 0.55). In contrast, the additional discriminant function with SRQ score explained the rest of the variance (8%, canonical correlation=0.19) and was not found to be statistically significant, as could be expected (Wilks’ Λ = .96, χ^2^(2) = 1.66, *p*>.05). The best fit discriminant function has the largest relationship with CFQ (standardized coefficient of.83), followed by participation diversity ((standardized coefficient of.67). Increase of 0.05 in cognitive failures and decrease of 0.08 in participation diversity increase together the probability to have a more severe illness symptoms. The discriminant function was found to classify correctly 88.5% of mild illness, 62.5% of moderate and marked illness severity, and only 6.7% of minimal illness with a general rate of correct classification of 59.2%.

## 4 Discussion

The severity of recent-onset mental health condition is a precursor of future illness course (4–7) and guides the clinical practice directed to ameliorate the long-term consequences of mental illness (1, 2). This study has addressed the general level of psychiatric symptomology to meet unique characteristics of recent illness onset. Being grounded in the staging and transdiagnostic approaches, the study focused on investigating the severity of recent-onset illness precursors aiming to address the challenge of early detection. This pilot study suggests a model to explain recent-onset illness severity across three levels: minor, mild, and moderate, and marked. However, it should be noted that this model is more sensitive to detecting more severe illness and may not effectively indicate minor illness. The extent of self-report on cognitive failures in everyday activities and limitations in the diversity of participation in daily life before the illness onset were found to be indicators of severity of recent-onset illness.

### 4.1 Cognition

Our findings add to the existing literature, demonstrating that the extent of premorbid cognitive impairments, as reported by the participants, is indicative of illness severity at an early stage. The report on cognitive errors or lapses in daily life situations, as measured with the CFQ, was sensitive for detecting the severity of illness. The representation of cognitive skills in daily life situations as reflected by the CFQ, aligns with the concept of functional cognition (47). Functional cognition has been indicated as an important target for measurement and health care, given its importance for positive health outcomes and everyday life in a range of populations (47); and it is one of the core targets for the evaluation and intervention by occupational therapists (47). Previous research on functional cognition in mental health has addressed mainly populations with prolonged mental illness, demonstrating its contribution to health status and functional outcomes (e.g., 48, 49). This study contributes to existing knowledge by demonstrating the importance of functional cognition for health as early as the initial phases of mental health conditions’ development. The findings further support the staging approach and underscores the relevance of occupational therapy theories and practices in this context. Within the recent onset of mental illness, the sensitivity of self-report on premorbid functional cognition to future illness severity can be further understood based on previous research on (1) the high prevalence of neurocognitive deficit among individuals at risk for the development of mental illness (15, 20), and (2) longer-term functional implications of neurocognitive impairments at onset (8).

Report on specific cognitive biases of “anomalous thinking” and “emotional reasoning” correlated with the illness severity index. The findings on the association of thinking patterns with illness severity are in line with the previous literature (16). Indeed, not-typical thinking patterns about the world and events has the potential to contribute to the symptoms’ formation in a range of mental health conditions (16, 21, 23, 25). Still, the Cognitive Biases Questionnaire was not specific enough to properly distinguish between the levels of severity. This suggests either a limited sensitivity of the tool or the possibility that the cognitive biases, as reported by the participants, are a general faculty in mental health conditions, rather than a specific attribute of some level of severity. In addition, the contradiction in the literature may stem from the timing of evaluation of cognitive biases across the studies: before the illness onset and formal diagnosis or after it; or, which type of cognitive bias was addressed: those occurring here and now versus premorbid patterns.

Additional findings highlight the importance of considering the timeline of evaluation. We found that current neurocognitive status was less indicative of the illness severity than premorbid neurocognitive functioning in daily life activities. Given that the modeling of the severity of recent illness onset is in its infancy, we suggest that this finding reveals the unique stage characteristics. First, a performance-based measure of neurocognitive status in the sub-acute stage, as was done in the current study, may still be less representative of the actual decline in cognition being blurred by the general context of coping with recent-onset mental illness. On the other hand, the self-report grounded on specific daily life occurrences may be a reliable source for gathering information on objective phenomena. Still, little congruency with findings on the role of the neurocognitive current status in the explanation of prolonged illness severity and emerging illness onset (7, 8, 15, 20) may stem from the research procedures. While most previous studies used comprehensive batteries with the potential to capture nuanced fluctuations in cognitive functions, this study involves a screening neurocognitive test only. In addition, the studies in prolonged illness addressed disability level, including independent living, employment status, or quality of life as an outcome measure (7, 36), rather than a general estimation of illness severity as was done in the present study.

### 4.2 Sensory modulation

To the best of our knowledge, this study was the first to investigate sensory modulation alterations in the context of recent-onset mental illness severity. Following previous studies with serious mental illness (27, 28), we found high rates of SMD in the population with recent illness onset, based on their self-report. Indeed, altered sensory modulation suggests living continuously with an aversive experience evoked by everyday typical, non-painful sensations or omitting important internal (body) signs and external (environment) information (31). Living with sensory alterations can lead to misinterpretations of reality. Consequently, individuals’ reactions to situations might be inappropriate according to social standards, which often fail to account for the invisible challenges of SMD. Alternatively, reactions may be socially acceptable but require individuals to endure discomfort. Both scenarios have a potential to be distressing and resource-intensive (27, 28). Indeed, it has been suggested that SMD may interfere with a coherent sense of self (50) and contribute to the formation of mental illness with symptoms of anxiety, avoidance, mood alteration, and even psychosis and dissociation (27, 28, 30, 50). The impact of SMD may be even more prominent given its involvement in additional processes, such as cognitive impairments. Proper cognitive processing requires intact input (51). In this light, SMD may contribute to cognitive failures and biases, such as the interpretation of events as threatening, as well as provoke catastrophic, attributional, and dichotomous thinking, interrupt reasoning and conclusion-making, and generally alter cognitive basic functions of attention (31, 52). Surprisingly, in this current study, self-report on SMD was not statistically distinctive within any of the illness severity groups neither in prevalence nor in specific type, even though effect size metrics suggested group difference trends. These findings imply that regardless of the mental illness severity level, SMD is a general attribute across mental health diagnoses. However, a prospective study that monitors sensory regulation processes throughout developmental stages until the onset of mental health conditions is required to deepen our understanding of the impact of SMD on the severity of recent illness onset.

### 4.3 Participation patterns

This study contributes to the existing literature by investigating participation in a range of areas of occupation through various activities by both objective and subjective dimensions before the mental illness onset based on self-report. Our findings expand previous reports on the contribution of premorbid employment, educational status, and general functional status to future illness trajectory (6, 14, 17). We demonstrated that lower participation diversity in a range of occupations including domestic life activities, learning and applying knowledge, and the scope of leisure activities, which precede the illness onset according with the participants report, is a reliable indicator of more severe illness. These findings further validate the importance of participation in occupations and activities for health and well-being (8, 9, 14, 33).

The particular difference between the groups of illness severity was found in recreation and leisure activities including, wandering around the home for leisure, visiting cultural and sports events, visiting family and friends, hosting, going out to dine, day trips, or overnight vacation. The findings are of importance since all these activities were recognized by individuals with mental illness in previous research as crucial for health, and they are in general under-attained (32, 33). The vulnerability of these activities to mental state fluctuations stems from their nature. These activities are non-obligatory and self-guided, requiring intrinsic motivation, self-organization, planning, and additional efforts to pursue, all these capacities were found to be affected by prolonged mental illness (3), at the onset of mental illness (8, 17), but also altered before the illness onset, as it was reported in the current study. Being indicative of mental health deterioration, recreation, and leisure activities have no recognized standards, posing difficulty in the detection of decline.

The findings of this pilot study suggest that additional differences between the groups in various participation indices may emerge in future research. This is evidenced by large effect size (53–55) in between-group differences in domestic life activities, learning and applying knowledge activities, and quiet leisure activities with the trend to be inferior in moderate illness as early as in the period preceding illness onset. Of note, these differences did not reach statistical significance and should be considered with caution, even though they are analogous to differences in prolonged stages of mental illness (7). Interpretation of effect size metrics expands our understanding of the discriminant quality of the participation diversity. From a developmental perspective, the possibility for a reduction in participation diversity is of particular concern among young adults, given the importance of leisure activities, learning, and applying knowledge, as well as practicing domestic life activities for personal formation and successful transition into adult life (33). Initial indications of between-group differences were seen in the report on a number of terminated activities in areas of quiet leisure, learning, and knowledge application, as well as in the level of enjoyment derived from participation in these areas. While the trend of difference between the groups was discerned solely through effect size metrics and was inconclusive, it does offer some insights. It hints that individuals with more severe mental illness tend to stop less quiet leisure activities, but this specific type of activity was less enjoyable for them. In addition, they may tend to discontinue learning and applying knowledge activities, which were more enjoyable for them. These patterns of particular concern give importance to participation in activities with health-promoting experience (32, 33, 35) and a high risk of becoming a constant participation pattern of disengagement from health-supportive activities and transition to the prolonged stage of the illness (14, 56). Still, this study failed to demonstrate a clear contribution of the subjective participation dimension to the distinguishing between the groups of illness severity.

Interestingly, despite the general low intensity/frequency of participation that was reported, no differences were found between the groups in this index. The results may stem from the sample size in each group. Still, they may suggest that before the onset of mental illness of any severity, people can keep the frequency of engagement, possibly at the expense of diversity. Alternatively, activities in which they maintain the involvement, predefine the frequency, and, thus, support the participation despite the changes preceding the illness onset. These findings support arguing for a need for a nuanced approach to the analysis of objective and subjective indices by areas of occupations to avoid misinterpretation of the participation patterns, and, thus, misidentification of the severity of recent-onset mental illness.

The model, proposed by this study, was not sensitive to minimal illness severity. Most of the individuals indicated by the staff with minimal illness were classified by the model with either mild or moderate levels of the illness. Three variables were enrolled in the model representing three concepts: functional cognition, everyday participation, and sensory modulation. These constructs were selected based on the knowledge of prolonged mental illness and transition into the illness, and initial statistical analysis. It may be that there are specific nuances of minimal illness versus other types of severity at illness onset, which are not prominent within existing literature, and/or were not captured by this study. For example, social cognition which has extensive evidence (24), and environmental factors were not addressed in the study. In addition, it may be that some of the measures, that were managed in the study (e.g., neurocognitive status screening tool), were not sensitive enough to differentiate between the groups. The implication of these findings will be further addressed in the conclusions. Additional intriguing findings were that, in contrast with the previous research, demographic factors of level of education, employment status, area of living, and living situation, were not indicative of the illness severity underscoring the uniqueness of this illness stage albeit certain similarities. In addition, no difference in the study variables of cognitive functioning, participation, and sensory modulation was found between two groups of major psychiatric diagnoses: psychotic and affective further supporting the transdiagnostic approach. Still, there was no difference in the illness severity by diagnostic groups.

### 4.4 Implications for occupational therapy

This study reveals that alterations in objective and subjective participation dimensions and reduced functional cognition may be hallmarks for those who is going to develop more serious mental illness. The study highlights the most sensitive dimensions and areas of participation, emphasizing the need for comprehensive evaluation and in-depth analysis. A warning sign is a decline in participation diversity, coupled with the extent of given-up activities, rather than changes in participation intensity. Additionally, a decrease in the level of enjoyment with participation may serve as a warning indicator. The most sensitive area of occupation across various dimensions is leisure entertainment activities. Two other occupational areas of concern, where a restriction in participation should be noted, are domestic life activities and learning and applying knowledge. These findings suggest that occupational therapy can provide pivotal information for the early identification of the most vulnerable populations by assessing these factors using tools developed within the profession. Therefore, occupational therapy should be an integrated part of the relevant teams.

### 4.5 Study limitations

The findings should be considered in light of the study’s limitations. The CGI was administrated by two clinicians from the research team. This method of administration enables standardization and reduces the risk of inter-rater variability, still it may be a source for biases within the rating due to limited knowledge of the participants and the potential impact of other tests’ results on the judgement. These biases may obscure the associations between the measurements. The sensitivity of the model to indicate minimal illness severity was negligible. Even though it is less constraining for clinical practice, given the importance of detecting the most severe illness, further research addressing additional factors with in-depth measurements is needed. The sample size was relatively small regarding the number of investigated parameters. The considerable variability among participants has posed challenges in achieving statistically significant differences between groups, with some differences being discerned solely through effect size metrics seriously limiting the strength of the findings. The groups of both the illness severity and diagnosis were unequal for the number of participants. Together these affect the overall discriminant quality of the model, which was, as had been reported, significant, but relatively low, being less indicative of minimal illness severity. Next, we used screening cognitive measurements limiting to the general evaluation of the constructs. Some additional factors that have the potential to contribute to the illness severity were eliminated by the research procedures (e.g., substance use) or unaddressed (e.g., childhood trauma), limiting the possibility to capture their impact. In addition, due to the characteristics of the investigated population, the data on participation, sensory modulation, cognitive failures, and biases were collected retrospectively through self-report, thus, its quality might be affected by timing and mental illness onset, and, it may represent early state of the illness rather than premorbid situation. Moreover, premorbid cognitive functioning was evaluated based on self-report. Subjective reporting can reflect self-perception of cognitive function rather the functioning itself. It is recommended to conduct a prospective study assessing participation, sensory modulation, and cognitive functioning before the onset of mental illness using both self-reported and performance-based measurements, followed by tracking illness severity after onset. This will help confirm the predictive quality of the proposed model and further expand our understanding of relevant factors and their sensitivity.

### 4.6 Conclusion

Early detection of more severe illness may be helpful for early intervention and prevention to ameliorate the long-term impact of mental illness on the individual and the whole community. This pilot study focused innovatively on the investigation of markers for the severity of recent-onset mental illness and contributed evidence for building prediction models. The findings support the Transdiagnostic approach in recent onset, as well as the staging approach. The study provides further support for the extent of cognitive biases and sensory modulation dysfunction, as was indicated based on self-report, in populations with recent onset suggesting their contribution to the illness formation. Still, the explanatory quality of these factors for recent illness severity has not been proven. The same is true for most demographic and illness-related factors, including diagnosis, part of them were previously established as associated with illness onset or its severity in prolonging stages. The most effective markers were found to be the level of functional cognition and limited diversity of participation in daily life activities, as were reported by the participants. The findings on the importance of an in-depth assessment of functional cognition and participation for the identification of a vulnerable population, delineate the unique role of occupational therapy within forces acting to mitigate the impact of serious mental illness. Moreover, the study offers initial empirical support for potential avenues for preventive interventions by occupational therapy, supporting the relevance of existing professional practices in prolonged mental illness for recent-onset stage. Still, further research is needed to expand our understanding of mechanisms to indicate and eliminate severe mental illness onset.

## Data Availability

The raw data supporting the conclusions of this article will be made available by the authors, without undue reservation.

## References

[1] Arango C Díaz-Caneja CM McGorry PD Rapoport J Sommer IE Vorstman JA. Preventive strategies for mental health. Lancet Psychiatr. (2018) 5:591–604. doi: 10.1016/S2215-0366(18)30057-9 29773478

[2] Worthington MA Cannon TD. Prediction and prevention in the clinical high-risk for psychosis paradigm: A review of the current status and recommendations for future directions of inquiry. Front Psychiatr. (2021) 12:770–4. doi: 10.3389/fpsyt.2021.770774 PMC8569129 34744845

[3] Galderisi S Heinz A Kastrup M Beezhold J Sartorius N. Toward a new definition of mental health. World Psychiatr. (2015) 14:231. doi: 10.1002/wps.v14.2 PMC4471980 26043341

[4] Burton CZ Tso IF Carrión RE Niendam T Adelsheim S Auther AM. Baseline psychopathology and relationship to longitudinal functional outcome in attenuated and early first episode psychosis. Schizophr Res. (2019) 212:157–62. doi: 10.1016/j.schres.2019.07.048 PMC6791749 31395490

[5] Hartmann JA Nelson B Ratheesh A Treen D McGorry PD. At-risk studies and clinical antecedents of psychosis, bipolar disorder and depression: a scoping review in the context of clinical staging. Psychol Med. (2019) 49:177–89. doi: 10.1017/S0033291718001435 29860956

[6] Lee RSC Hermens DF Scott J O’Dea B Glozier N Scott EM. A transdiagnostic study of education, employment, and training outcomes in young people with mental illness. Psychol Med. (2017) 47:2061–70. doi: 10.1017/S0033291717000484 28393749

[7] Harvey PD Strassnig MT Silberstein J. Prediction of disability in schizophrenia: Symptoms, cognition, and self-assessment. J Exp Psychopathol. (2019) 10:2043808719865693. doi: 10.1177/2043808719865693

[8] Crouse JJ Chitty KM Iorfino F Carpenter JS White D Nichles A. Modelling associations between neurocognition and functional course in young people with emerging mental disorders: a longitudinal cohort study. Transl Psychiatry. (2020) 10:1–9. doi: 10.1038/s41398-020-0726-9 32066687 PMC7026055

[9] de Nijs J Burger TJ Janssen RJ Kia SM van Opstal DP de Koning MB. Individualized prediction of three-and six-year outcomes of psychosis in a longitudinal multicenter study: a machine learning approach. NPJ Schizoph. (2021) 7:34. doi: 10.1038/s41537-021-00162-3 PMC8253813 34215752

[10] Lee LHN Procyshyn RM White RF Gicas KM Honer WG Barr AM. Developing prediction models for symptom severity around the time of discharge from a tertiary-care program for treatment-resistant psychosis. Front Psychiatr. (2023) 14:1181740. doi: 10.3389/fpsyt.2023.1181740 PMC10282838 37350999

[11] Van Os J Reininghaus U. Psychosis as a transdiagnostic and extended phenotype in the general population. World Psychiatr. (2016) 15:118–24. doi: 10.1002/wps.20310 PMC4911787 27265696

[12] Harrison LA Kats A Williams ME Aziz-Zadeh L. The importance of sensory processing in mental health: A proposed addition to the research domain criteria (RDoC) and suggestions for RDoC 2.0. Front Psychol. (2019) 10:103. doi: 10.3389/fpsyg.2019.00103 30804830 PMC6370662

[13] Kotov R Krueger RF Watson DA. paradigm shift in psychiatric classification: the hierarchical taxonomy of psychopathology (HiTOP). World Psychiatr. (2018) 17:24–5. doi: 10.1002/wps.20478 PMC5775140 29352543

[14] Iorfino F Hermens DF Shane PM Zmicerevska N Nichles A Badcock CA. Delineating the trajectories of social and occupational functioning of young people attending early intervention mental health services in Australia: a longitudinal study. BMJ Open. (2018) 8:e020678. doi: 10.1136/bmjopen-2017-020678 PMC5875606 29588325

[15] Caspi A Houts RM Ambler A Danese A Elliott ML Hariri A. Longitudinal assessment of mental health disorders and comorbidities across 4 decades among participants in the Dunedin birth cohort study. JAMA Netw Open. (2020) 3:e203221–e203221. doi: 10.1001/jamanetworkopen.2020.3221 32315069 PMC7175086

[16] Torgalsbøen AK Mohn C Rund BR. Neurocognitive predictors of remission of symptoms and social and role functioning in the early course of first-episode schizophrenia. Psychiatry Res. (2014) 216:1–5. doi: 10.1016/j.psychres.2014.01.031 24530159

[17] Cotter J Drake RJ Bucci S Firth J Edge D Yung AR. What drives poor functioning in the at-risk mental state? A systematic review. Schizophr Res. (2014) 159:267–77. doi: 10.1016/j.schres.2014.09.012 25261041

[18] American Occupational Therapy Association. Occupational therapy practice framework: Domain and process (4th ed.). Am J Occup Ther. (2020) 74:7412410010. doi: 10.5014/ajot.2020.74S2001 34780625

[19] World Health Organization. ICD-11: International Classification of Diseases (11th revision) (2022). Available online at: https://icd.who.int/ (Accessed at January 21, 2024).

[20] Bora E Pantelis C. Meta-analysis of cognitive impairment in first-episode bipolar disorder: comparison with first-episode schizophrenia and healthy controls. Schizophr Bull. (2015) 41:1095–104. doi: 10.1093/schbul/sbu198 PMC4535631 25616505

[21] Wright AC Davies G Fowler D Greenwood KE. Self-defining memories predict engagement in structured activity in first episode psychosis, independent of neurocognition and metacognition. Schizophr Bull. (2019) 45:1081–91. doi: 10.1093/schbul/sby155 PMC6737466 30388257

[22] Peters ER Moritz S Schwannauer M Wiseman Z Greenwood KE Scott J. Cognitive biases questionnaire for psychosis. Schizophr Bull. (2014) 40:300–13. doi: 10.1093/schbul/sbs199 PMC3932080 23413104

[23] Gaweda L Prochwicz K. A comparison of cognitive biases between schizophrenia patients with delusions and healthy individuals with delusion-like experiences. Eur Psychiatr. (2015) 30:943–9. doi: 10.1016/j.eurpsy.2015.08.003 26647870

[24] Pinkham AE Penn DL Green MF Buck B Healey K Harvey PD. The social cognition psychometric evaluation study: results of the expert survey and RAND panel. Schizophr Bull. (2014) 40:813–23. doi: 10.1093/schbul/sbt081 PMC4059426 23728248

[25] Villalobos D Pacios J Vázquez C. Cognitive control, cognitive biases and emotion regulation in depression: a new proposal for an integrative interplay model. Front Psychol. (2021) 12:628416. doi: 10.3389/fpsyg.2021.628416 33995183 PMC8119761

[26] Ludwig KA Pinkham AE Harvey PD Kelsven S Penn DL. Social cognition psychometric evaluation (SCOPE) in people with early psychosis: A preliminary study. Schizophr Res. (2017) 190:136–43. doi: 10.1016/j.schres.2017.03.001 PMC5735418 28302395

[27] Brown C Karim R Steuter M. Retrospective analysis of studies examining sensory processing preferences in people with a psychiatric condition. Am J Occup Ther. (2020) 74:7404205130p1–7404205130p11. doi: 10.5014/ajot.2020.038463 32602452

[28] van den Boogert F Klein K Spaan P Sizoo B Bouman YH Hoogendijk WJ. Sensory processing difficulties in psychiatric disorders: A meta-analysis. J Psychiatr Res. (2022) 151:173–80. doi: 10.1016/j.jpsychires.2022.04.020 35489177

[29] Bar-Shalita T Seltzer Z Vatine JJ Yochman A Parush S. Development and psychometric properties of the Sensory Responsiveness Questionnaire (SRQ). Disabil Rehabil. (2009) 31:189–201. doi: 10.1080/09638280801903096 18608365

[30] Javitt DC. Sensory processing in schizophrenia: neither simple nor intact. Schizophr Bull. (2009) 35:1059–64. doi: 10.1093/schbul/sbp110 PMC2762632 19833806

[31] Schmitt CM Schoen S. Interoception: A multi-sensory foundation of participation in daily life. Front Neurol. (2022) 16:875200. doi: 10.3389/fnins.2022.875200 PMC9220286 35757546

[32] Hodgekins J French P Birchwood M Mugford M Christopher R Marshall M. Comparing time use in individuals at different stages of psychosis and a non-clinical comparison group. Schizophr Res. (2015) 161:188–93. doi: 10.1016/j.schres.2014.12.011 25541138

[33] Thomas EC Snethen G Salzer MS. A developmental study of community participation of individuals with serious mental illnesses: Implications for policy and practice. Am J Orthopsychiatry. (2017) 87:597. doi: 10.1037/ort0000269 28394152

[34] Imms C Adair B Keen D Ullenhag A Rosenbaum P Granlund M. [amp]]lsquo;Participation’: a systematic review of language, definitions, and constructs used in intervention research with children with disabilities. Dev Med Child Neurol. (2016) 58:29–38. doi: 10.1111/dmcn.12932 26411643

[35] Elstad TA Johannsen GS. Mental health, participation and social identity. In: Participation in Health and Welfare Services, (New York, NY: Routledge) (2017). p. 156–69.

[36] Zimmerman M Morgan TA Stanton K. The severity of psychiatric disorders. World Psychiatr. (2018) 17:258–75. doi: 10.1002/wps.20569 PMC6127765 30192110

[37] Guy W. ECDEU Assessment manual for psychopharmacology. NIMH. (1976). doi: 10.1037/e591322011-001

[38] Berk M Ng F Dodd S Callaly T Campbell S Bernardo M. The validity of the CGI severity and improvement scales as measures of clinical effectiveness suitable for routine clinical use. J Eval Clin Pract. (2008) 14:979–83. doi: 10.1111/j.1365-2753.2007.00921.x 18462279

[39] Busner J Targum SD. The clinical global impressions scale: applying a research tool in clinical practice. Psychiatry (Edgmont). (2007) 4:28–37. PMC2880930 20526405

[40] Bar-Shalita T Deutsch L Honigman L Weissman-Fogel I. Ecological aspects of pain in sensory modulation disorder. Res Dev Disab. (2015) 45:157–67. doi: 10.1016/j.ridd.2015.07.028 26254166

[41] Nasreddine ZS Phillips NA Bedirian V Charbonneau S Whitehead V Collin I. The Montreal Cognitive Assessment, MoCA: A brief screening tool for mild cognitive impairment. J Am Geriatr Soc. (2005) 53:695–9. doi: 10.1111/j.1532-5415.2005.53221.x 15817019

[42] Broadbent DE Cooper PF FitzGerald P Parkes KR. The cognitive failures questionnaire (CFQ) and its correlates. Br J Clin Psychol. (1982) 21:1–16. doi: 10.1111/j.2044-8260.1982.tb01421.x 7126941

[43] Carrigan N Barkus E. A systematic review of cognitive failures in daily life: Healthy populations. Neurosci Biobehav Rev. (2016) 63:29–42. doi: 10.1016/j.neubiorev.2016.01.010 26835660

[44] Wallace JC Kass SJ Stanny CJ. The cognitive failures questionnaire revisited: dimensions and correlates. J Gen Psychol. (2002) 129:238–56. doi: 10.1080/00221300209602098 12224809

[45] Jarus T Barnea R Vaserlaf N Bortz L Yakyal S Gal-On E. The development of participation questionnaire for adult Israeli population. Isr J Occup Ther. (2006) 15:93–111.

[46] Sherry A. Discriminant analysis in counseling psychology research. Couns Psychol. (2006) 34:661–83. doi: 10.1177/0011000006287103

[47] Giles GM Edwards DF Baum C Furniss J Skidmore E Wolf T. Making functional cognition a professional priority. Am J Occup Ther. (2020) 74:7401090010p1–7401090010p6. doi: 10.5014/ajot.2020.741002 PMC7018454 32078504

[48] Scanlan JN Still M. Functional profile of mental health consumers assessed by occupational therapists: Level of independence and associations with functional cognition. Psychiatry Res. (2013) 208:29–32. doi: 10.1016/j.psychres.2013.02.032 23521900

[49] Lipskaya-Velikovsky L Jarus T Kotler M. Prediction of the intensity and diversity of day-to-day activities among people with schizophrenia using parameters obtained during acute hospitalization. Disabil Rehabil. (2017) 39:1300–6. doi: 10.1080/09638288.2016.1194896 27346369

[50] Sass LA Parnas J. Schizophrenia, consciousness, and the self. Schizophr Bull. (2003) 29:427–44. doi: 10.1093/oxfordjournals.schbul.a007017 14609238

[51] Palmer SE Kimchi R. The information processing approach to cognition. In: Knapp TJ Robertson LC, editors. Approaches to Cognition: Contrasts and Controversies. Lawrence Erlbaum Associates, Hillsdale, NJ (1986). p. 37–77.

[52] Ramsay IS Schallmo MP Biagianti B Fisher M Vinogradov S Sponheim SR. Deficits in auditory and visual sensory discrimination reflect a genetic liability for psychosis and predict disruptions in global cognitive functioning. Front Psychiatr. (2020) 11:638. doi: 10.3389/fpsyt.2020.00638 PMC7358403 32733293

[53] Lakens D. Calculating and reporting effect sizes to facilitate cumulative science: a practical primer for t-tests and ANOVAs. Front Psychol. (2013) 4:863. doi: 10.3389/fpsyg.2013.00863 24324449 PMC3840331

[54] Funder DC Ozer DJ. Evaluating effect size in psychological research: Sense and nonsense. AMPPS. (2019) 2:156–68. doi: 10.1177/2515245919847202

[55] Schuele CM Justice LM. The importance of effect sizes in the interpretation of research: Primer on research: Part 3. ASHA Leader. (2006) 11:14–27. doi: 10.1044/leader.FTR4.11102006.14

[56] Eklund M Argentzell E. Perception of occupational balance by people with mental illness: A new methodology. Scand J Occup Ther. (2016) 23:304–13. doi: 10.3109/11038128.2016.1143529 26872496

